# Simultaneous Analysis of Organic Acids, Glycerol and Phenolic Acids in Wines Using Gas Chromatography-Mass Spectrometry

**DOI:** 10.3390/foods13020186

**Published:** 2024-01-05

**Authors:** Violeta Garcia-Viñola, Candela Ruiz-de-Villa, Jordi Gombau, Montse Poblet, Albert Bordons, Cristina Reguant, Nicolas Rozès

**Affiliations:** 1 Grup de Biotecnologia Microbiana dels Aliments, Departament de Bioquímica i Biotecnologia, Facultat d’Enologia, Universitat Rovira i Virgili, c/Marcel·lí Domingo s/n, 43007 Tarragona, Catalonia, Spain; violeta.garcia@urv.cat (V.G.-V.); candela.ruiz@urv.cat (C.R.-d.-V.); montserrat.poblet@urv.cat (M.P.); 2 Grup de Tecnologia Enològica, Departament de Bioquímica i Biotecnologia, Facultat d’Enologia, Universitat Rovira i Virgili, c/Marcel·lí Domingo s/n, 43007 Tarragona, Catalonia, Spain; jordi.gombau@urv.cat; 3 Grup de Biotecnologia Enològica, Departament de Bioquímica i Biotecnologia, Facultat d’Enologia, Universitat Rovira i Virgili, c/Marcel·lí Domingo s/n, 43007 Tarragona, Catalonia, Spain; albert.bordons@urv.cat (A.B.); cristina.reguant@urv.cat (C.R.)

**Keywords:** tartaric acid, fumaric acid, pyruvic acid, sorbic acid, glyoxylic acid, oxaloacetic acid, glycerol, gallic acid, ferulic acid, caffeic acid

## Abstract

Fermented beverages, particularly wines, exhibit variable concentrations of organic and phenolic acids, posing challenges in their accurate determination. Traditionally, enzymatic methods or chromatographic analyses, mainly high-performance liquid chromatography (HPLC), have been employed to quantify these compounds individually in the grape must or wine. However, chromatographic analyses face limitations due to the high sugar content in the grape must. Meanwhile, phenolic acids, found in higher quantities in red wines than in white wines, are typically analyzed using HPLC. This study presents a novel method for the quantification of organic acids (OAs), glycerol, and phenolic acids in grape musts and wines. The approach involves liquid-liquid extraction with ethyl acetate, followed by sample derivatization and analysis using gas chromatography-mass spectrometry (GC-MS) in selected ion monitoring (SIM) detection mode. The results indicated successful detection and quantification of all analyzed compounds without the need for sample dilution. However, our results showed that the method of adding external standards was more suitable for quantifying wine compounds, owing to the matrix effect. Furthermore, this method is promising for quantifying other metabolites present in wines, depending on their extractability with ethyl acetate. Fermented beverages, particularly wines, exhibit variable concentrations of organic and phenolic acids, posing challenges in their accurate determination. Traditionally, enzymatic methods or chromatographic analyses, mainly high-performance liquid chromatography (HPLC), have been employed to quantify these compounds individually in the grape must or wine. The approach of this proposed method involves (i) methoximation of wine compounds in a basic medium, (ii) acidification with HCl, (iii) liquid-liquid extraction with ethyl acetate, and (iv) silyl derivatization to analyze samples with gas chromatography-mass spectrometry (GC-MS) in ion monitoring detection mode (SIM). The results indicated successful detection and quantification of all analyzed compounds without the need for sample dilution. However, our results showed that the method of adding external standards was more suitable for quantifying wine compounds, owing to the matrix effect. Furthermore, this method is promising for quantifying other metabolites present in wines, depending on their extractability with ethyl acetate. In other words, the proposed method may be suitable for profiling (targeted) or fingerprinting (untargeted) strategies to quantify wine metabolites or to classify wines according to the type of winemaking process, grape, or fermentation.

## 1. Introduction

Organic acids (OAs) contribute to the chemical composition and organoleptic properties of wine and improve its microbiological and physicochemical stability [1]. Some OAs, such as L-tartaric, L-malic, and citric acids, are mainly found in the grape must, while others are produced during fermentative processes, such as alcoholic (AF) and malolactic (MLF) fermentations and processes such as ageing or stabilization of wines [2]. Among the OAs produced during AF and MLF, the most important are succinic, oxaloacetic, α-ketoglutaric, and fumaric acids, which are involved in the tricarboxylic acid (TCA) cycle, as well as D- and L-lactic acids, pyruvic acid, and citramalic acid from the metabolism of yeasts and lactic acid bacteria [3]. Other organic acids are considered additives, and their use is authorized by the Organisation Internationale de la vigne et du vin (OIV). This is the case for sorbic acid, which is used in the food industry in the form of potassium sorbate for its antimicrobial effect, especially in yeast, and fumaric acid, which has recently been authorized by the OIV at a concentration of 0.6 g/L in wines [4] as an acidifier and for its inhibitory effect on lactic bacteria responsible for MLF [5]. Generally, the concentrations of the abovementioned OAs depend on a variety of factors, such as the grape must variety, fermentation conditions, including presence of oxygen, and yeasts or bacteria used, which causes certain difficulties for their quantification because the concentration ranges of these acids are wide in the must or wine. For instance, tartaric or malic acid can be found at levels above 5000 mg/L and citric acid at a level of 1000 mg/L, while other acids of the TCA cycle can be found at a concentration of approximately 30 mg/L [6]. In this context, it seems difficult to find a generic analytical method allowing the identification and quantification of OAs under different conditions. Glycerol, a byproduct of AF (glyceropyruvic fermentation by yeast), is also found in wines, with a concentration of up to 3 g/L, depending on the type of vinification and physicochemical conditions of AF.

In addition, phenolic compounds are present in the grape must and wine, and they can be divided into two groups: the family of nonflavonoids, such as benzoic and hydroxycinnamic acids, and stilbenes, and flavonoid compounds, such as anthocyanins, flavan-3-ols, and flavonols [7]. The concentrations of phenolic compounds or phenol carboxylic acids are mainly influenced by the grape variety, i.e., white or red grapes, but also by the stage of maturity and the type of vinification, such as skin maceration of the grapes [8]. Some phenolic compounds, such as benzoic and hydroxycinnamic acids, are involved in the oxidation phenomena in grape musts and can exhibit antibacterial, antimutagenic, or anti-inflammatory activity [8]. Most of these phenolic acids (PAs) are substrates for the polyphenol oxidase enzymes present in the grape must and wine, such as tyrosinase from the grape must and laccase from *Botrytis cinerea*, which are responsible for the browning of white wines [9]. This is especially noticeable in white musts, in which the content of phenolic compounds is lower than that in red grapes. In addition, these PAs can serve as substrates for certain yeasts, causing significant organoleptic modifications in wines and the production of ethyl phenols by *Brettanomyces* [10]. In this sense, some strains of *Saccharomyces cerevisiae*, with cinnamate decarboxylase activity, can promote the formation of vinyl phenols via decarboxylation of these phenolic acids. Vinyl phenols, in turn, can be reduced to ethyl phenols by *Brettanomyces* or can react with anthocyanins in red wine to form a new family of pigments, called pyranoanthocyanins [11].

Analysis of OAs, glycerol, and phenolic acids is rather complicated because of their high diversity and varying concentration ranges in wines. To our knowledge, there is no chromatographic method able to simultaneously analyze these three classes of compounds in wine. The determination of OAs, glycerol, and PAs has been the subject of numerous methodological developments in recent years, thanks to advancements in the technology of chromatographic analysis. Enzymatic analysis methods are widely used in the food industry [12]. These methods allow precise quantification but are for single use only; in other words, the specificity of enzymes implies that the main OAs present in the grape must or wines are quantified one by one. Although devices can be used to automate enzymatic reactions and help reduce the time consumed, enzymatic kits do not exist for all compounds; for instance, tartaric acid is determined via a colorimetric method using a vanadium salt in acidic environments. Moreover, no enzymatic method allows the quantification of phenolic acids in wines. Meanwhile, methods of chromatographic analysis have progressed, especially liquid or gas chromatography coupled with mass spectrometry (LC-MS and GC-MS, respectively). Thus, the main OAs in grape juices and wines are often determined via high-performance liquid chromatography (HPLC), capillary electrophoresis (CE), GC-MS, or Fourier-transform infrared spectroscopy (FTIR) analysis [13], and, more recently, using amperometric biosensors for the classification of wines based on carboxylic acid levels [14]. According to [6], the main analytical method used for the determination and quantification of OAs in wines was an HPLC technique in approximately 50% of published cases.

On the other hand, sample preparation can be a limitation for the analysis of complex matrices. Typically, wine samples do not require additional preparation steps, such as filtration, centrifugation, or solid phase extraction (SPE), to be analyzed. However, for instance, grape juice, due to its high sugar content, may need to be diluted before analysis or extracted by an organic solvent such as ethyl acetate in order to reduce sugar interference in later steps of sample preparation before GC-MS analysis. Nowadays, some alternatives such as SPE can be used to improve the selectivity of compounds of interest to be analyzed from biological samples or complex matrices. In this sense, there are different commercial SPE cartridges [15,16] for the isolation of analytes. As far as we are concerned, representative applications of SPE as a sample preparation tool in food analysis mainly focus on the analysis of pesticides [17], phenolic compounds of wines [15], edible oils [18], etc. In all cases, when using any SPE sorbent, the operator must rinse and equilibrate the resins before loading the samples, which can also be conditioned depending on the nature of the compounds that one wishes to extract, either by acidification or by basification. Another disadvantage of using SPE for the isolation of a large family of compounds is the economic cost and time consumed compared to conventional liquid/-liquid extraction (LLE) with an organic solvent. Thus, when comparing SPE and LLE techniques for analyzing organic acids in urine samples, using the SPE procedure increases the average number of organic acids detected compared to LLE without significantly improving accuracy [19]. These authors concluded that the cost of LLE treatment was economical and could be a practical option if used for analysis of organic acids.

Using chromatographic techniques, alcohols such as ethanol and glycerol can be separated from OAs and their levels determined. On the other hand, to our knowledge, there is no HPLC method allowing the analysis of OAs at the same time as phenolic acids. PAs cannot be separated using the same columns as those used for OAs, and moreover, the use of a UV-Vis photodiode array (PDA) detector is recommended for the detection of PAs [20]. However, in foods other than wine, some research has shown that it is possible to use a GC-MS method to separate and identify benzoic and phenolic acids, such as their trimethylsilyl derivatives, at very different amounts in cranberries [21]. On the other hand, GC-MS techniques are increasingly used in studies of the endometabolome or metabolic fingerprinting of cells, tissues, and organisms [22,23] and in the analysis of exometabolomic or metabolic footprinting [24] of liquid samples, such as human urine for the early detection of disease [25] or wines [26]. Regarding the method developed by [26] for wines, GC-MS analysis can be used simultaneously for the determination of monosaccharides, amino acids (AAs), and OAs. This method allows the determination of 2 hexoses, 13 OAs, and 13 AAs after methoximation (use of methoxyamine) and silylation (use of N-methyl-N-trimethylsilyltrifluoroacetamide, MSTFA) using ribitol as an internal standard. Although this method is reliable for examining the chemical composition of wines, it is not suitable for determining OAs in grape juices because of their high sugar content, which can saturate the column response, and for determining phenolic acids in wines.

The aim of this study was to propose a new method for the determination of organic acids, glycerol, and phenolic acids in samples as complex and different as wines. The derivatization of this type of samples after extraction with ethyl acetate made it possible to quantify these compounds. The proposed method is not limited to OAs, glycerol, and PAs, which were studied in this work; many other compounds from grape musts and wines, such as alcohol, acid, and keto functional groups, could also be derived via silylation and therefore identified using data libraries. Thus, acids, such as glyceric, 2-isopropylmalic, and glutaric acids, as well as alcohols, such as 2,3-butanediol, tyrosol, or 2-phenylethanol, were also detected. In other words, the proposed method could be suitable for profiling (targeted) or fingerprinting (untargeted) strategies to quantify wine metabolites or to classify wines according to the type of winemaking process, grape, or fermentation.

## 2. Materials and Methods

### 2.1. Materials

#### 2.1.1. Reagents

All organic acids and the silylation reagent (N-methyl-N-trimethylsilyltrifluoroacetamide, MSTFA) were purchased from Sigma-Aldrich (Barcelona, Spain). The composition of standard organic acids (OAs) and phenolic acids (PAs) was prepared from concentrated solutions of lactic acid, glyoxylic acid, potassium sorbate, pyruvic acid, succinic acid, fumaric acid, sodium citramalate, L-malic acid, α-ketoglutaric acid, L-tartaric acid, citric acid, cinnamic acid, vanillic acid, shikimic acid, syringic acid, p-coumaric acid, gallic acid, ferulic acid, and caffeic acid, and the sodium salt of D-gluconic acid was used as a standard additive in white wine. Glycerol (Panreac, Barcelona, Spain) was also included in the mixed solution. Tridecanoic acid (C13; Sigma-Aldrich) was used as an internal standard. All compounds were diluted in ultrapure Milli-Q water, except sodium oxaloacetate, which was prepared in a solution of 0.1 M HCl [27]. For the extraction of these wine compounds, different solvents were preliminarily tested, namely ethyl acetate and methyl tert-butyl ether (MTBE) from Panreac and diethyl ether from SDS (Peypin, France), to determine the most effective extraction solvent.

#### 2.1.2. Wines

Two wines, Muscat of Alexandria white wine and a red wine aged in oak barrels (blend of Cabernet Sauvignon, Merlot, and Tempranillo wines) from the experimental cellar Mas dels Frares at the Faculty of Enology of the Rovira i Virgili University of Tarragona (Spain), were used to determine recoveries via the incremental addition of authentic standards of organic acids, glycerol, and phenolic acids.

### 2.2. Methods

#### 2.2.1. Sample Preparation

To prepare a standard solution of organic and phenolic acids, a mixture of twelve organic acids, glycerol, and eight phenolic acids at various concentrations was prepared in ultrapure Milli-Q water. The wines used for external additions were previously centrifuged. Briefly, the procedure described in Table 1 was as follows: 400 μL of a wine sample or a standard (calibration curve) plus 10 μL of C13 (internal standard at 1.28 g/L diluted at 50% (*v*/*v*) in ethanol) were used for analysis. The concentration ranges of all the compounds analyzed are shown in Table 2.

This protocol is used for the derivatization of mixed compounds (keto acids, organic acids, and alcohols) and includes oximation of keto groups to prevent enolization or chemical loss and trimethylsilylation (TMS) of labile hydrogens on polar compounds. In our case, oximation was performed with 2.5% (*w*/*v*) hydroxylamine in ultrapure Milli-Q water [28] because the chemical reaction with this reagent was better than that with a methoxyamine hydrochloride solution. Before two ethyl acetate extractions, samples were acidified with 6 N HCl. Then, MSTFA was used for the TMS process of the compounds after drying the ethyl acetate extract at 45 °C for 40 min in a vacuum centrifuge (Thermo Fisher Scientific, Waltham, MA, USA).

For the evaluation of the best extraction solvent, results for 2 × 400 μL of ethyl acetate were compared with those for (i) 1 × 400 μL of diethyl ether + 1 × 400 μL of ethyl acetate and (ii) 1 × 400 μL of MTBE + 1 × 400 μL of ethyl acetate.

#### 2.2.2. Chromatographic Conditions

Three microliters of a derivatized sample was injected in split (1:10) mode into a 6890 N GC system (Agilent Technologies, Waldbronn, Germany) equipped with a DB-5HT column (30 m × 0.25 mm × 0.1 μm; Agilent Technologies). Helium was used as the carrier gas at a constant flow of 1.0 mL/min. The compounds were detected with a mass selective detector (MSD; Model 5975, Agilent Technologies). The MSD temperatures were 300 °C, 150 °C, and 250 °C for the transfer, quadrupole, and source, respectively. The MSD data were acquired in electronic ionization scan mode at 70 eV within a 35–650 amu range after a solvent delay of 3.50 min and then analyzed using the Agilent MSD Chemstation software (Agilent Technologies). Metabolites were identified using an in-house library and the NIST 2017 library. For peak integration, a set of characteristic and selective fragments (SIM detection mode) was chosen for each compound (Table S1) according to [29]. The relative abundance of each identified compound was calculated according to the respective chromatographic peak area corrected to the IS (C13) peak area. The results were expressed as normalized areas.

#### 2.2.3. Linearity Range and Limits of Detection and Quantification

To determine the linearity range of the method, 11 concentrations of each compound were processed as explained above (Section 2.2.1). Using the areas obtained with the SIM detection method for each compound, normalization was performed by dividing the area of the compound by that of the internal standard (C13). A total of 10 replicates for each concentration were analyzed, and from the averages of these replicates, a least-squares linear regression curve was calculated.

The limits of detection (LOD) and quantification (LOQ) were calculated based on the standard deviation (SD) of the response and the slope obtained from the linear regression curve for low concentrations [30]. The following equations were used:

- LOD (mg) = 3.3 × RMSE/slope of the curve;
- LOQ (mg) = 10 × RMSE/slope of the curve.

The root-mean-square error (RMSE) was determined from the regression curve.

#### 2.2.4. Recovery and the Matrix Effect

The accuracy of the analysis was established using the standard addition method. Specifically, three intermediate concentrations of a mixture of OAs and PAs were chosen and added to the two different matrices, white wine and aged red wine. The recovery of compounds was calculated by plotting the concentrations calculated with the equations established from the standard calibration curves in water in Table 2 against the theoretical concentrations. The slopes of least-squares linear regression curves for each compound added to the two wines allowed us to determine their recoveries from the two different matrices according to [31].

To calculate the response to the external addition of standards, the method described by [32] was used. In short, for a straight-line equation, the x-intercept is -b/m, where b is the intercept and rm is the slope. The uncertainty or standard deviation of the x-intercept was calculated according to [32], taking into account the covariance.

#### 2.2.5. Comparison of Our GC Method with Other Analytical Methods

To compare the performance of the proposed method with that of other analytical methods, certain OAs and PAs in the white and red wines (see preparation of different wines in Section 2.1.2.) were analyzed. Glycerol and tartaric acid were measured enzymatically and with a colorimetric method, respectively, on a Y15 enzymatic autoanalyzer (BioSystems S.A., Barcelona, Spain) using kits from BioSystems [33]. For PAs, p-coumaric acid and ferulic acid were determined with HPLC with an Agilent 1200 series liquid chromatograph (HPLC-diode array detection) using an Agilent Zorbax Eclipse XDB-C18 column (4.6 × 250 mm, 5-μm; Agilent Technologies) according to a modification of the method of [20].

### 2.3. Statistical Analysis

All analyses were performed using three independent biological samples. Data were statistically analyzed using principal component analysis, ANOVA, and Tukey’s test, performed using the XLSTAT 2020.2.3 software (Addinsoft, Paris, France). Statistical significance was considered at a *p* value < 0.05. Least-squares linear regression was used to construct the calibration curves of the standards. Principal component analysis was used in a preliminary study to determine the best extraction solvent. Additionally, the RMSE was calculated to compare the accuracy of different methods, with the lowest RMSE value indicating the best accuracy.

## 3. Results

### 3.1. Effects of Extraction Solvents

***Preliminary studies***. To choose the best extraction solvent for organic acids, glycerol, and phenolic acids and an adequate volume of sample for analysis, several preliminary investigations were carried out. Thus, three types of liquid-liquid extractions, according to [25,34], were performed using volumes of 200 and 400 μL of aged red wine. The sample derivatization procedure was the same as described in Table 1. In other words, hydroxylamine and MSTFA were used for the oximation and silylation of all compounds, respectively. In previous tests, methoxyamine was used for the methoximation of compounds, but both the sample preparation time and methoxyamine effectiveness for the methoximation of certain ketoacids were not adequate. By analyzing the peak areas normalized by the area of C13, 54 metabolites were used with an identification quality greater than 70% according to the NIST17 library (Table S1). The results of the principal component analysis (PCA) showed that the variables that contributed the most to a positive correlation with both components were found on the right side of the PCA plot (Figure S1B) and, therefore, implicitly indicated which method of compound extraction would be the most suitable for wine samples. It could be deduced that one of the best extraction procedures was using ethyl acetate 2 times. Indeed, on the right side of the PCA plot, wine samples extracted with 400 μL of ethyl acetate (A4) and 1 × ethyl acetate or 1 × MTBE (C4) seemed to be the best choices (Figure S1A). Unlike the findings of [25,35], the addition of saturated NaCl before or after the oximation or acidification procedure did not improve the extraction of metabolites from wine samples. For this reason, no addition of NaCl was made before the extraction. Finally, two extractions with 400 μL of ethyl acetate were chosen for the rest of the study.

### 3.2. Detection of Organic and Phenolic Acids

**Figure 1:**
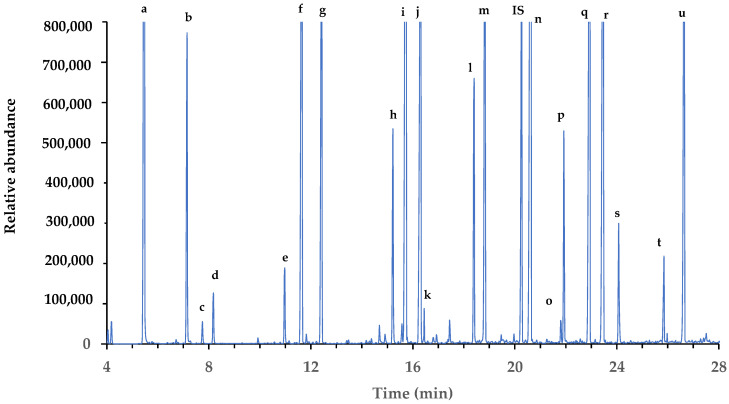
Chromatogram of organic acids, glycerol, and phenolic acids from a standard mixture. Peaks: a, lactic acid; b, glyoxylic acid; c, pyruvic acid; d, sorbic acid; e, glycerol; f, succinic acid; g, fumaric acid; h, citramalic acid; i, malic acid; j, cinnamic acid; k, oxaloacetic acid; l, α-ketoglutaric acid; m, tartaric acid; IS, tridecanoic acid (C13); n, vanillic acid; o, shikimic acid; p, citric acid; q, syringic acid; r, p-coumaric acid; s, gallic acid; t, ferulic acid; u, caffeic acid.

***Establishment of calibration curves for all compounds in the water matrix***. Based on the results obtained above, a mixture of organic acids (12), glycerol, and phenolic acids (8) was produced in a wide concentration range (Table 2) for their identification using an internal MS standard and the NIST 2017 library and to obtain the best ion identifiers (Table S2). As shown in Figure 1, all organic acids, glycerol, and phenolic acids studied at intermediate concentrations were well separated within 28 min on the chromatogram.

### 3.3. Linearity

The linearity of the method was established by preparing eleven concentrations of each organic acid, glycerol, and nine concentrations for phenolic acids in ultrapure Milli-Q water, covering the expected concentration ranges in grape musts and wines (Table 2). For example, for major OAs, such as malic and tartaric acids, the maximum level for the calibration curve was 7 g/L; for all PAs, this was 0.2 g/L. The calibration point of each compound was injected twice from the same concentration range, and five extraction procedures were performed. Finally, ten calibration points were established for each compound (5 extraction procedures × 2 injections = 10 points). The normalization of the peak area for each compound was carried out to the area of C13 (internal standard, IS). This fatty acid was used as an IS because its retention time appeared in the middle of the total duration of the chromatographic program, which made it possible to minimize possible errors during sample preparation and those due to chromatographic conditions (column wear, injection, etc.). Calibration curves were constructed from the average of all concentrations of the mixture of standard solutions. Thus, the calibration curve for each compound was established by plotting the normalized area of the compound versus concentration. To the curve, least-squares linear regression was applied. In Table 2, a summary of these curve equations, concentration ranges, and coefficients of determination of the linear regression for each analyzed compound is presented. All the coefficients of determination were greater than 0.99.

### 3.4. Limits of Detection (LOD) and Quantification (LOQ)

The LOD and LOQ of each compound were calculated from the five lowest concentrations (Table 2) of the solutions in ultrapure Milli-Q water. Regarding the limits of detection (LOD) and quantification (LOQ), the proposed method allowed us to measure these compounds at low levels. Thus, the LODs ranged from 0.58 to 41.07 mg/L, and the LOQs ranged from 1.74 to 124.44 mg/L (Table 2). These lowest and highest values were estimated for glyoxylic acid and glycerol, respectively. These LODs and LOQs seem to be adequate for the determination of these compounds because their contents are much higher in wines. Indeed, it would not be a problem to quantify them.

### 3.5. Recovery

This section covers recoveries of compounds from white and red wine matrices. First, the accuracy of the method was investigated through recovery experiments of metabolites spiked into samples of the white wine and aged red wine. Method recovery was calculated by plotting the concentrations calculated with the equations in Table 2 from the normalized compound area (compound area/C13 area) for each spiked wine concentration against the theoretical concentrations. The slopes of the linear regression curves for each compound added to the two wines allowed the determination of their recoveries from the two different matrices according to [31]. According to [36], a satisfactory sample recovery is estimated to be between 70 and 130%. In our case, as shown in Table 3, some OAs could be perfectly quantified as their percentages of recovery varied from 60 to 111%. These were glyoxylic, pyruvic, succinic, fumaric, citramalic, α-ketoglutaric, and citric acids, the maximum levels of which in wines can reach 1 g/L. On the other hand, recoveries were less optimal for compounds present at higher levels in wines, such as glycerol and tartaric, malic, and lactic acids, which can reach concentrations up to 6–7 g/L, depending on the maturity of the wine grape, the fermentation process, or the type of winemaking. These recoveries ranged from 16% (tartaric acid in the white wine) to 262% (malic acid in the white wine).

Although the concentration of oxaloacetic acid in wine is generally low, the percentage recovery of this acid was noticeably low (Table 3). This decrease could be attributed to the inherent instability of oxaloacetic acid, leading to its rapid degradation during wine storage [27].

**Figure 2:**
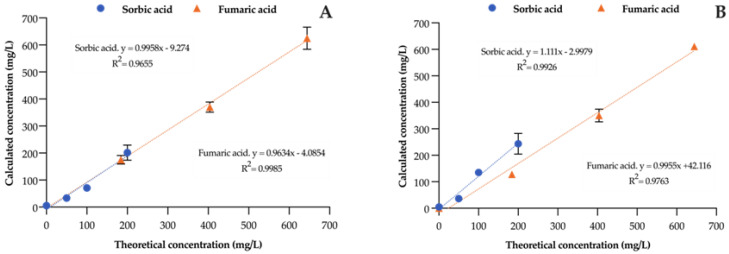
Recovery of sorbic acid and fumaric acid from white wine (**A**) and aged red wine (**B**). The *Y*−axis shows the concentrations calculated from the equations in Table 2.

For instance, as shown in Figure 2, recoveries of sorbic and fumaric acids, which are used as additives to wine to inhibit yeasts or lactic acid bacteria, ranged from 96 to 111%. Specifically, for sorbic acid, the concentrations ranged from 50 to 200 mg/L, while for fumaric acid, the range of the added concentrations was 200 to 600 mg/L.

Concerning the recovery of glycerol from the wines, significantly higher values were obtained (Table 3). In this instance, the concentrations of glycerol ranged from 100 to 400 mg/L, and glycerol values in the order of 58 and 32% were obtained. In other words, the linearity was good, but the ability to quantify this compound in the matrix wines based on the water calibration curve was not satisfactory. Another reason for the low recovery is that the partition coefficient of glycerol in the biphasic ethyl acetate/hydroalcoholic extraction solvent was used.

In the case of phenolic acids, concentrations between 10 and 50 mg/L were added. The recovery percentages for cinnamic, vanillic and syringic acids were satisfactory, ranging from 77% to 157%. However, in the case of shikimic acid, the recovery percentages for this phenolic acid were the lowest, 58% from the white wine and 2% from the aged red wine. On the other hand, for gallic, p-coumaric, ferulic, and caffeic acids, the recovery percentages were higher than 143%. These results may be due to interferences in the matrix or to the instability of the compounds, which may influence analyte recovery [37].

In summary, the recoveries of OAs and PAs were always higher from the red wine than from the white wine. This fact could be linked to the greater chemical complexity of red wines. The presence of phenolic acids in the form of anthocyanins, proanthocyanidins, etc., in red wine could modify the composition of the extract during sample preparation.

***Evaluation of the matrix effect.*** Additionally, another approach was used to calculate the recoveries and concentrations of compounds in the samples. Intermediate concentrations of a mixture of OAs and PAs (the same ones as in Table 2) were chosen and added to the white wine and aged red wine. However, instead of calculating the concentrations of the compounds according to the equations established in water (Table 2), we constructed linear regression curves from the normalized peak areas of the compounds relative to the theoretical concentrations. The results presented in Table 4 show that the coefficients of determination for all the studied compounds were appropriate, ranging from 0.9808 (lactic acid in the white wine) to 0.9998 (p-coumaric acid in the white wine).

The results obtained using the equations in Table 4 for all the compounds made it possible to estimate their concentrations in the two wines. For example, the lactic acid content was higher in the aged red wine than in the white wine due to malolactic fermentation, which may be related to the total malic acid consumption in aged red wine.

On the other hand, some of these compounds had concentrations lower than the corresponding LOD and LOQ determined in water (Table 2). Generally, the lowest concentrations were encountered in the white wine rather than in the aged red wine, particularly for phenolic acids (Table 4). Additionally, the results for glycerol and tartaric acid in both wines were very low. We found 12.6 and 1674 mg/L glycerol in the white wine and aged red wine, respectively, and an average of 760 mg/L tartaric acid in both wines. Faced with this incongruity, we had to increase the quantities of these two compounds in the two wines to better approximate their true concentrations. For this, the three intermediate additions were 0.552, 1.104, and 1.66 g/L for the two compounds. The results showed that after adjusting the additions of the two components to these wines, the concentrations were closer to those that can be found in white and red wines. For glycerol, the concentrations were 3.46 ± 0.28 and 5.67 ± 0.31 g/L for the white wine and aged red wine, respectively. For tartaric acid, they were 1.81 ± 0.32 and 3.02 ± 0.21 g/L, respectively.

However, another consideration can be made by examining the results in Table 4. The standard deviations calculated for the concentrations of all the compounds studied were adequate. In other words, the coefficient of variation of each compound was generally acceptable, showing that the external addition of standards was suitable.

### 3.6. Comparison of the Performance of the Proposed Method and Other Methods

To compare the contents of certain compounds obtained with the proposed method with those obtained via other methods, we analyzed the same white wine and aged red wine using the method of external addition of standards. For this comparison, the wines were spiked with increasing concentrations of glycerol, tartaric acid, p-coumaric acid, and ferulic acid. In these enriched samples, the compound contents were determined via GC-MS, enzymatic, colorimetric, and HPLC-DAD analyses. The results are shown in Table 5.

The results indicated that in the case of the additions to the white wine, no significant differences (*p* values > 0.05) were observed between the GC-MS and colorimetric methods for tartaric acid and the HPLC and GC-MS methods for p-coumaric and ferulic acids. The only difference was observed between the GC-MS and enzymatic methods for glycerol, with a *p* value of 0.016. However, in the case of the red wine, significant differences could be observed in the quantification of glycerol and the two phenolic acids (Table 5). Nevertheless, for tartaric acid, no significant differences were found (*p* value = 0.0992).

These results suggest that this method would be valid for the detection and quantification of organic and phenolic acids in white wine. Furthermore, with regard to red wine, other considerations could be taken into account, such as a more precise adjustment of external additions for certain compounds and the analysis of other red wines. Regardless, enzymatic and HPLC methods also have their limits in terms of precision. For the enzymatic determination of glycerol, samples must be diluted 1/10, which can lead to certain errors. For the determination of phenolic acids by HPLC, red wine samples must be conditioned via column separation before HPLC injection, and losses may therefore occur.

## 4. Discussion

Organic acids (OAs), glycerol, and phenolic acids are constituents of grape musts and wines, due to the intrinsic composition of the former and to fermentation processes in the presence of microorganisms for the latter. The OAs found in wines are not only important for their organoleptic impact but also have potential health benefits. From a human health perspective, OAs present in wines are considered to have weak antioxidant activity; however, they can promote iron absorption [37] and protect from the development of diabetes [38]. On the other hand, some OAs can inhibit the growth of yeasts and lactic acid bacteria during the production of wines. Sorbic, fumaric, and succinic acids are used to microbiologically stabilize wines during bottling to avoid organoleptic alterations. Furthermore, among these intrinsic compounds of the grape must and wine, phenolic acids exhibit antibacterial [39], antioxidant [40], anticarcinogenic, and anti-inflammatory activities [8]. For these reasons, it is important to determine the chemical composition of grape musts and wines to evaluate the concentration balance between OAs and predict the chemical evolution of wines and, consequently, their microbiological sensitivity during storage.

The chemical composition of wines is very complex, both in terms of the concentration ranges and organic structures, and its determination involves the use of different analytical methods. Most of these methods use HPLC systems connected to UV or MS detectors to separately quantify OAs and PAs. Other analytical methods, such as enzymatic methods, are very specific and precise but involve individual determination of compounds, and moreover, not all analytical possibilities are available on the market. In this context, the proposed method offers certain guarantees to identify and determine numerous compounds of interest present in white and red wines.

We finally targeted 12 OAs, which included intermediates of the TCA cycle as well as some OAs present in wines, such as tartaric, glyoxylic, citramalic, and lactic acids, and some enological additives, such as sorbic and fumaric acids. Additionally, glycerol, as a side product of glyceropyruvic fermentation, was incorporated in this study. Finally, eight PAs present in grape musts, and in particular red wines or skin maceration wines, were determined.

According to our preliminary results, the use of ethyl acetate twice is sufficient and practical to extract the compounds from the wine and to also avoid contamination with the sugars present in wines, which are insignificantly extracted with this solvent. Unfortunately, gluconic acid was not detected under these extraction conditions up to a concentration of 2 g/L in wines.

The results showed that the LOD and LOQ calculated for our method were higher than those determined for different analytical methods by different authors (Table 3) but are satisfactory considering the levels of compounds present in wines. Furthermore, in terms of validation parameters (linearity, concentration range, LOD, LOQ, and repeatability of sample injection over several days) [32], the newly proposed method is suitable when standard compounds are dissolved in ultrapure Milli-Q water.

On the other hand, it was impossible to determine the efficiency of extraction of the compounds because of the intrinsic property of the method, namely the use of hydroxylamine for the oximation of keto acids, the best method according to [28], before extraction with ethyl acetate. Thus, improving the extraction protocol before and after the addition of standards was impossible.

Unfortunately, when wine samples, whether white wine or aged red wine, were analyzed, the calculated concentrations of many wine compounds were not accurate, owing to the matrix effects of the two wines analyzed. The recovery proved to be unsuitable, particularly in the cases of phenolic acids and certain organic acids, such as tartaric acid, present in large quantities in wines, as well as for glycerol and red wine, due to the weakness of the extraction with ethyl acetate. For this reason, the external addition of the most representative standards of the determined matrices, in this case wines, may be necessary to quantify the compounds. Under these conditions, according to [39], the best way to quantify compounds in a complex matrix is to use the method of the external addition of standards.

On the other hand, other compounds were identified but not quantified using the NIST17 library, with an identification quality greater than 70%. Glyceric acid, 2-isopropylmalic acid or 2-IPMA, an intermediate of leucine biosynthesis in yeast [40], and glutaric acid, as well as alcohols such as 2,3-butanediol, an alternative metabolite of pyruvate or tyrosol, were also detected.

## 5. Conclusions

The proposed method is suitable for determining the main organic acids, glycerol, and phenolic acids in wines using GC-MS in SIM detection mode. However, to obtain robust results, the quantification of these compounds must be carried out by adding external standards because of the complexity of the matrices used, white or red wine. To do this, it is important to adjust the range of OA concentrations. In other words, for glycerol, lactic, malic, and tartaric acids, and, possibly, citric acid, the external addition must approximately reach the concentration expected in wines. The chemical composition of wines is very complex and very variable, and this method can also be useful in SCAN detection mode for establishing wine profiles.

## Figures and Tables

**Table 1:** Description of the method. *, Q.S., quantity sufficient for.

	Sample (μL)	Standard (μL)
Internal Standard (C13)	10	10
Sample	400	(10 to 400)
Ultrapure Milli-Q water	-	Q.S.* 400
30 μL of 30% (*w*/*v*) NaOH in ultrapure Milli-Q water		
80 μL of 2.5% (*w*/*v*) hydroxylamine-HCl in ultrapure Milli-Q water		
60 °C, 30 min		
80 μL of 6 N HCl in ultrapure Milli-Q water 2 × 400 μL of ethyl acetate		
Sample dried for 40’ under vacuum at 45 °C		
50 μL of MTSFA		
70 °C, 30 min		
Injection of 3 μL into the GC-MS system		

**Table 2:** Limit of detection (LOD) and Limit of quantification (LOQ), concentration range, linear regression equation, and R^2^ (determination coefficient) of organic acids, glycerol, and phenolic acids.

Compounds	LOD (mg/L)	LOQ (mg/L)	Concentration Range (mg/L)	Linear Regression Equation	R^2^
Lactic acid	8.20	24.84	2.69–4292	y = 0.0044x + 0.4166	0.9939
Glyoxylic acid	0.58	1.74	0.38–600	y = 0.0242x − 0.0119	0.9984
Pyruvic acid	2.85	8.63	0.90–718	y = 0.0007x + 0.0084	0.9933
Sorbic acid	7.14	21.64	0.20–312	y = 0.0126x − 0.0399	0.9955
Glycerol	41.07	124.44	4.46–8600	y = 0.0009x − 0.0914	0.9916
Succinic acid	1.43	4.33	0.81–1287	y = 0.0195x + 0.2219	0.9958
Fumaric acid	4.16	12.61	0.72–1147	y = 0.0315x + 0.0705	0.9964
Citramalic acid	1.88	5.70	0.30–474	y = 0.0099x + 0.0485	0.9973
Malic acid	11.98	36.31	3.11–4976	y = 0.0048x + 0.4983	0.9907
Cinnamic acid	6.76	20.48	0.69–228	y = 0.0242x − 0.0385	0.9980
Oxaloacetic acid	2.51	7.61	0.68–540	y = 0.0131x + 0.0112	0.9997
α-Ketoglutaric acid	1.03	3.11	0.33–519	y = 0.0225x + 0.0765	0.9938
Tartaric acid	5.87	17.77	4.42–7042	y = 0.0018x − 0.0076	0.9990
Vanillic acid	3.50	10.61	0.59–193	y = 0.0392x + 0.0372	0.9966
Shikimic acid	30.13	91.31	5.00–200	y = 0.0019x − 0.0198	0.9924
Citric acid	11.80	35.76	0.76–1207	y = 0.0035x + 0.0305	0.9980
Syringic acid	5.37	16.27	0.74–243	y = 0.0366x − 0.3418	0.9921
p-Coumaric acid	8.16	24.72	0.72–238	y = 0.0305x − 0.0441	0.9989
Gallic acid	5.82	17.62	0.75–252	y = 0.0084x − 0.0814	0.9915
Ferulic acid	9.29	28.16	0.57–189	y = 0.0040x − 0.0075	0.9985
Caffeic acid	1.39	4.21	0.75–248	y = 0.0184x − 0.0432	0.9967

**Table 3:** Equations and coefficient of determination of organic acids, glycerol, and phenolic acids calculated from the additions of external standards using the equations in Table 2. Recoveries were deduced from the slope × 100, (*n* = 3 for each addition of compound used).

	White Wine			Aged Red Wine		
Compounds	Equation	R^2^	% Recovery	Equation	R^2^	% Recovery
Lactic acid	y = 4.8462x + 310.93	0.9519	485	y = 9.2146x + 6625.7	0.9533	921
Glyoxylic acid	y = 0.8179x + 4.1975	0.983	82	y = 0.5982x + 5.8733	0.9804	60
Pyruvic acid	y = 1.0722x + 37.524	0.9932	107	y = 1.7071x + 17.13	0.9927	170
Sorbic acid	y = 0.9958x − 9.274	0.9655	99	y = 1.111x − 2.9979	0.9926	111
Glycerol	y = 0.5778x + 364.03	0.9657	58	y = 0.3196x + 849.95	0.9503	32
Succinic acid	y = 1.5125x + 477.31	0.9917	151	y = 1.5164x + 952.79	0.9868	152
Fumaric acid	y = 0.9634x − 4.0854	0.9985	96	y = 0.9955x + 42.116	0.9763	99
Citramalic acid	y = 1.061x + 0.6636	0.9982	106	y = 1.4497x + 5.7235	0.9877	145
Malic acid	y = 2.262x + 886.46	0.9929	262	y = 1.3465x − 35.462	0.9806	135
Cinnamic acid	y = 0.769x + 1.792	0.9855	77	y = 0.7845x + 5.3769	0.9256	78
Oxaloacetic acid	y = 0.2647x − 0.1312	0.999	26	y = 0.0368x + 9.3218	0.8198	4
α-Ketoglutaric acid	y = 0.9588x + 12.697	0.9947	96	y = 0.8811x + 33.45	0.9963	88
Tartaric acid	y = 0.1639x + 290.13	0.932	16	y = 1.477x + 3143.3	0.9791	148
Vanillic acid	y = 0.9177x + 1.2922	0.9802	92	y = 1.1262x + 7.347	0.9524	113
Shikimic acid	y = 0.5808x + 41.308	0.3349	58	y = 0.0250x − 0.0680	0.5273	2
Citric acid	y = 1.2404x + 279.13	0.9896	124	y = 1.4104x + 31.261	0.9914	141
Syringic acid	y = 0.816x + 9.287	0.9947	82	y = 1.5691x + 22.363	0.938	157
p-Coumaric acid	y = 1.4311x + 10.695	0.9981	143	y = 1.6319x + 66.095	0.9028	163
Gallic acid	y = 4.7908x + 5.476	0.9905	479	y = 8.9203x + 277.96	0.9949	892
Ferulic acid	y = 8.491x + 50.09	0.9957	849	y = 10.43x + 61.19	0.9540	1043
Caffeic acid	y = 2.9775x + 6.211	0.9955	298	y = 5.8921x + 43.071	0.9849	589

**Table 4:** Equations and coefficient of determination of linear regression, and concentrations (mg/L) of organic acids, glycerol, and phenolic acids using the method of external standard additions. *, concentration (mg/L) of standard addition. (*n* = 3 for each concentration).

	White Wine			Aged Red Wine		
Compounds	Equation	R^2^	Concentration	Equation	R^2^	Concentration
Lactic acid * 52; 105; 210	y = 0.0206x + 1.1354	0.9808	65.7 ± 7.7	y = 0.0907x + 25.043	0.9942	276.1 ± 9.1
Glyoxylic acid 12.15; 24.3; 48.6	y = 0.0195x + 0.1105	0.9931	5.7 ± 0.9	y = 0.0145x + 0.1617	0.9938	11.2 ± 0.9
Pyruvic acid 28.8; 57.6; 115.2	y = 0.0034x + 0.0109	0.9955	3.3 ± 1.5	y = 0.0031x + 0.269	0.9937	87.5 ± 3.6
Sorbic acid 34; 68; 136	y = 0.0243x + 0.482	0.9948	19.8 ± 2.2	y = 0.0134x − 0.0325	0.9956	2.4 ± 1.6
Glycerol 102; 204; 408 552; 1104; 1656	y = 0.0029x + 0.0363 y = 0.042x + 0.0121	0.9939 0.9846	12.6 ± 5.5 3460 ± 280	y = 0.0046x + 7.6979 y = 0.067x + 0.012	0.987 0.9711	1673.5 ± 66.9 5667 ± 313
Succinic acid 49.7; 99.4; 198.8	y = 0.0352x + 9.2003	0.9933	261.4 ± 9.2	y = 0-0356x + 18.754	0.9933	526.8 ± 16.0
Fumaric acid 33.2; 66.4; 132.8	y = 0.0389x − 0.0235	0.9912	0.6 ± 0.2	y = 0.0388x + 0.0394	0.9904	1.0 ± 0.2
Citramalic acid 25.4; 50.8; 101.6	y = 0.0121x + 0.023	0.9965	2.1 ± 1.1	y = 0.015x + 0.0449	0.9943	3.0 ± 1.5
Malic acid 105; 210; 420	y = 0.0123x + 4.2333	0.9918	344.2 ± 15.8	y = 0.0076x + 0.1612	0.9910	21.2 ± 7.8
Cinnamic acid 11.4; 22.8; 45.6	y = 0.0201x + 0.017	0.9976	0.08 ± 0.03	y = 0.0208x + 0.1251	0.9982	6.01 ± 0.40
Oxaloacetic acid 15; 30; 60	y = 0.0037x − 0.0008	0.9981	5.6 ± 2.5	y = 0.0022x + 01258	0.9845	55.5 ± 3.4
α-Ketoglutaric acid 25.5; 51; 102	y = 0.0215x + 0.3624	0.9952	16.9 ± 1.6	y = 0.023x + 0.8483	0.9976	38.0 ± 1.4
Tartaric acid 102.5; 205; 410	y = 0.0103x + 8.1485	0.9918	789.6 ± 28.3	y = 0.0077x + 5.5463	0.9908	722.5 ± 27.8
552; 1104; 1656	y = 0.0031x + 5.7264	0.9931	1807 ± 320	y = 0.0032x + 9.7186	0.9717	3019 ± 210
Vanillic acid 9.7; 19.4; 38.8	y = 0.0377x + 0.0844	0.9903	2.24 ± 0.63	y = 0.0394x + 0.3522	0.9957	8.94 ± 0.52
Citric acid 50.5; 101; 202	y = 0.0043x + 1.0243	0.9926	237.1 ± 9.2	y = 0.005x + 0.1555	0.9971	31.4 ± 2.4
Syringic acid 12.2; 24.4; 48.6	y = 0.03x + 0.0714	0.9962	2.38 ± 0.49	y = 0.0429x + 0.7869	0.9949	18.35 ± 0.83
p-Coumaric acid 11.9; 23.8; 47.6	y = 0.053x + 0.1340	0.9981	2.54 ± 0.34	y = 0.0570x + 2.2733	0.9876	39.91 ± 1.89
Gallic acid 12.6; 25.2; 50.4	y = 0.0164x + 0.1751	0.9946	10.67 ± 0.74	y = 0.0709x + 2.0484	0.9983	30.51 ± 0.62
Ferulic acid 9.4; 18.8; 37.6	y = 0.03751x + 0.10054	0.9936	2.68 ± 0.63	y = 0.0269x + 0.1944	0.9929	7.23 ± 0.75
Caffeic acid 12.4; 24.8; 49.6	y = 0.0315x + 0.1202	0.9939	3.82 ± 0.65	y = 0.0925x + 0.9057	0.9969	9.79 ± 0.54

**Table 5:** Comparison of methods for determining the content of glycerol, tartaric acid, p-coumaric acid, and ferulic acid in white wine and aged red wine. The external standard addition method was used for enzymatic, colorimetric, HPLC, and GC analyses. Concentrations were calculated from the Harris equation using the least-squares linear regression method.

	White Wine		Aged Red Wine	
	Enzymatic	GC	Enzymatic	GC
**Glycerol**	4.04 ± 0.47	3.46 ± 0.28	10.47 ± 0.20	5.67 ± 0.31
***p*-value**	0.016		<0.0001	
	**Colorimetric**	**GC**	**Colorimetric**	**GC**
**Tartaric acid**	1.92 ± 0.09	1.81 ± 0.32	3.13 ± 0.07	3.02 ± 0.21
***p*-value**	0.2607		0.0992	
	**HPLC**	**GC**	**HPLC**	**GC**
**p-Coumaric acid**	2.11 ± 1.26	2.54 ± 0.34	1.30 ± 0.69	39.91 ± 1.89
***p*-value**	0.1975		<0.0001	
**Ferulic acid**	2.59 ±0.82	2.68 ± 0.63	2.64 ± 0.23	7.23 ± 0.75
***p*-value**	0.7302		<0.0001	

## Data Availability

Data is contained within the article or Supplementary Materials.

## Supplementary Materials

The following supporting information can be downloaded at: https://www.mdpi.com/article/10.3390/foods13020186/s1, Table S1: List of 54 compounds used for PCA analysis identified by the NIST 17 library from aged red wine. Identification quality > 70%. Table S2: Characteristic ions and retention times of TMS derivatized organic acids, glycerol, phenolic acids and internal standard (tridecanoic acid) used for peak integration in SIM detection method according to [29]. Figure S1: Graphs of the effect of extraction solvent on profiles of aged red wines obtained by hydroxylamine and MSTFA derivatization (A) and variable loadings (B). A, 2 × ethyl acetate; B, 1 × ethyl acetate + 1 × diethyl ether; C, 1 × ethyl acetate + 1 × MTBE. 2200 μL; 4400 μL.
