# Supplementary material for: Simultaneous Analysis of Organic Acids, Glycerol and Phenolic Acids in Wines Using Gas Chromatography-Mass Spectrometry
Source: Foods. 2024 Jan 5;13(2):186. doi: 10.3390/foods13020186 (PMC10814861; doi:10.3390/foods13020186)
Supplement: Supplementary file 1 [file foods-13-00186-s001.zip › foods-2781928-supplementary.pdf]

Supplementary Materials.

Table S1. List of 54 compounds used for PCA analysis identified by the NIST 17 library from aged red wine. Identification quality > 70%.

Number	Compounds	Number	Compounds
1	2,3-Butanediol 1	28	3-hydroxydodecanoic acid
2	2,3-Butanediol 2	29	Oxaloacetic acid
3	1,3-Propanediol	30	Tyrosol
4	Lactic acid	31	2-Isopropylmalic acid
5	3-hydroxyisovaleric acid	32	Unknown 5
6	Unknown 1	33	1,2,3-ButaneTriol
7	Glyoxylic acid	34	4-hydroxybenzoic acid
8	2-Furoic acid	35	[illegible]-ketoglutaric acid
9	1,4-Butanediol	36	3,3-dimethyl glutaric acid
10	Pyruvic acid	37	Tartaric acid
11	4-hydroxybutanoic acid	38	Phloretic acid
12	2-hydroxyvaleric acid	39	Vanillic acid
13	Sorbic acid	40	2,6-dihydroxybenzoic acid
14	Glyoxime	41	Protocatechuic acid
15	2-Phenylethanol	42	Citric acid
16	1-Propanol	43	Isocitric acid
17	Hexanoic acid	44	Unknown 6
18	Unknown 2	45	Syringic acid
19	Methyl ethyl Succinic acid	46	4-hydroxy phenyllactic acid
20	Unknown 3	47	4-Coumaric acid
21	Octanoic acid	48	Ethyl Gallate
22	Glycerol	49	Shikimic acid
23	Succinic acid	50	Gallic acid
24	2-Pentanol	51	Palmitic acid
25	Unknown 4	52	Caffeic acid
26	Citramalic acid	53	4-Hydroxyphenyllactic acid
27	Malic acid	54	Stearic acid

Table S2. Characteristic ions and retention times of TMS derivatized organic acids, glycerol, **phenolic acids** and internal standard (tridecanoic acid) used for peak integration in SIM detection method according to [29] (Roessner et al., 2000).

Chromatogram number	Compounds	Retention time (min)	Characteristic ions (*m/z*)
**1**	Lactic acid	5.455	117, 133, 147, 191, 219, 234
**2**	Glyoxylic acid	7.141	73, 147, 190, 218, 233
**3**	Pyruvic acid	7.753	75, 95, 125, 151, 169, 184
**4**	Sorbic acid	8.186	73, 147, 214, 233, 261, 304
**5**	Glycerol	10.984	73, 103, 117, 133, 147, 205
**6**	Succinic acid	11.651	73, 129, 147, 247, 262
**7**	Fumaric acid	12.429	83, 133, 143, 147, 245
**8**	Citramalic acid	15.229	73, 115, 147, 247, 259, 349
**9**	Malic acid	15.724	73, 133, 147, 233, 245, 335
**10**	**Cinnamic acid**	**16.311**	**77, 103, 131, 161, 205, 220**
**11**	Oxaloacetic acid	16.452	73, 89, 142, 184, 276, 333
**12**	a-Ketoglutaric acid	18.411	73, 147, 156, 198, 229, 288
**13**	Tartaric acid	18.831	73, 147, 189, 219, 292, 423
**14**	Tridecanoic acid (IS)	20.288	73, 117, 129, 145, 271, 286
**15**	**Vanillic acid**	**20.636**	**73, 223, 267, 297, 312**
**16**	**Shikimic acid**	**21.823**	**73, 147, 204, 255, 372, 462**
**17**	Citric acid	21.944	147, 273, 347, 363, 375, 465
**18**	**Syringic acid**	**22.949**	**73, 253, 297, 312, 327, 342**
**19**	**p-Coumaric acid**	**23.484**	**73, 179, 219, 249, 293, 308**
**20**	**Gallic acid**	**24.098**	**73, 281, 355, 443, 458**
**21**	**Ferulic acid**	**25.870**	**73, 249, 293, 308, 323, 338**
**22**	**Caffeic acid**	**26.664**	**103, 147, 205, 292, 319, 333**

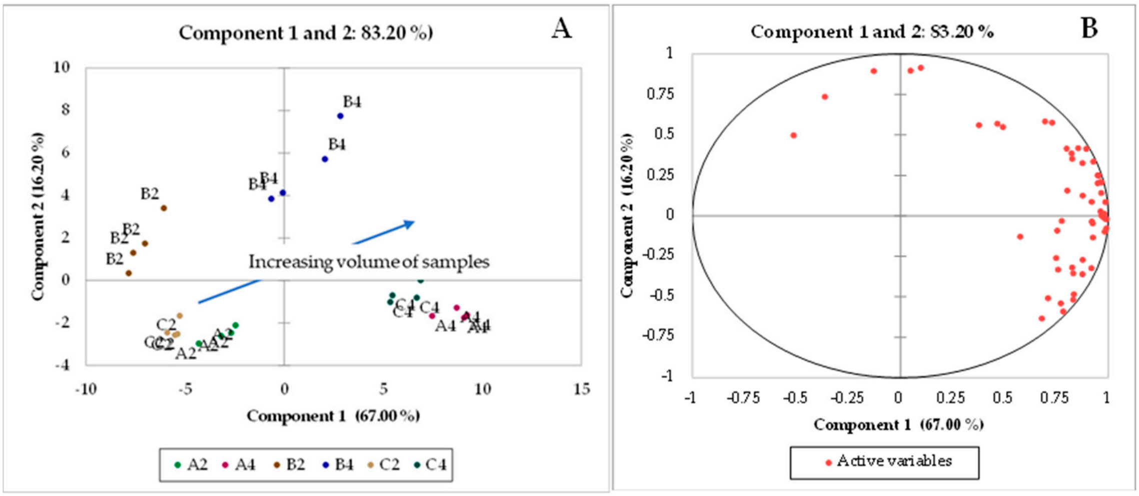

Figure S1. Graphs of the effect of extraction solvent on profiles of aged red wines obtained by hydroxylamine and MSTFA derivatization (A) and variable loadings (B). A, 2 x ethyl acetate; B, 1 x ethyl acetate + 1 x diethyl ether; C, 1 x ethyl acetate + 1 x MTBE. 2, 200 µl; 4,400 µl.

29 Roessner, U.; Wagner, C.; Kopka, J.; Trethewey, R.N.; Willmitzer, L. Simultaneous analysis of metabolites in potato tuber by gas chromatography-mass spectrometry. *Plant J.* **2000**, *23*, 131–142. https://doi.org/10.1046/j.1365-313x.2000.00774.x.
